# A library of sensitive position-specific scoring matrices for high-throughput identification of nuclear pore complex subunits

**DOI:** 10.1093/nargab/lqad025

**Published:** 2023-03-23

**Authors:** Andreas N Ioannides, Katerina R Katsani, Christos A Ouzounis, Vasilis J Promponas

**Affiliations:** Bioinformatics Research Laboratory, Department of Biological Sciences, University of Cyprus, Nicosia 1678, Cyprus; Department of Molecular Biology & Genetics, Democritus University of Thrace, Alexandroupolis 68100, Greece; Artificial Intelligence & Information Analysis Lab, School of Informatics, Aristotle University of Thessalonica, Thessalonica 54124, Greece; Chemical Process & Energy Resources Institute, Centre for Research & Technology Hellas, Thessalonica 57001, Greece; Bioinformatics Research Laboratory, Department of Biological Sciences, University of Cyprus, Nicosia 1678, Cyprus

## Abstract

The nuclear pore complex exhibits different manifestations across eukaryotes, with certain components being restricted to specific clades. Several studies have been conducted to delineate the nuclear pore complex composition in various model organisms. Due to its pivotal role in cell viability, traditional lab experiments, such as gene knockdowns, can prove inconclusive and need to be complemented by a high-quality computational process. Here, using an extensive data collection, we create a robust library of nucleoporin protein sequences and their respective family-specific position-specific scoring matrices. By extensively validating each profile in different settings, we propose that the created profiles can be used to detect nucleoporins in proteomes with high sensitivity and specificity compared to existing methods. This library of profiles and the underlying sequence data can be used for the detection of nucleoporins in target proteomes.

## INTRODUCTION

**Figure 1.**
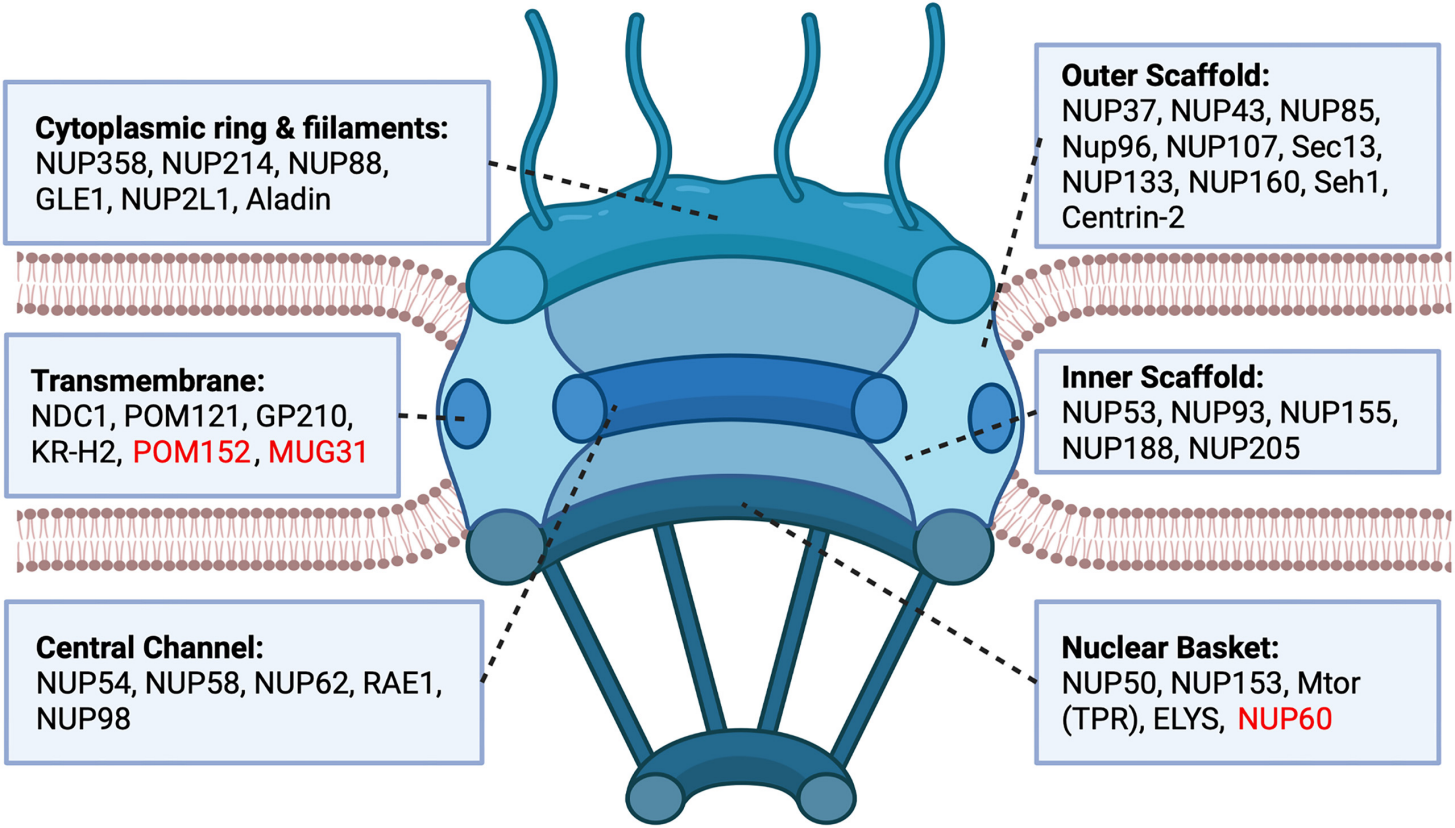
The human nuclear pore complex and its major subcomplexes. Proteins colored with red are found only in fungal species. Created with Biorender.com.

Eukaryotic cells contain membrane-bound organelles, including the nucleus, which is enclosed by the nuclear envelope, a double lipid bilayer membrane that contains the genetic material, along with a number of proteins. The double membranated nuclear envelope is impermeable to large molecules, while macromolecular transport is regulated by a multiprotein complex, called the Nuclear Pore Complex (NPC) (1). Diffusion of ions and small molecules, with size smaller than 9 nm, is enabled (2); it has been suggested that the NPC can be dilated up to ∼39 nm to allow passage of larger molecules (3). The nuclear pore structure research goes back to the late 60s, where it was discovered that the nuclear envelope contains openings along its surface (4). Later studies have shown that the aforementioned ‘openings’ on the surface of the nucleus are pores constructed by a multiprotein complex (5–9). Nuclear pore complexes are among the largest macromolecular structures in a eukaryotic cell (90–120 MDa in human cells) and the majority of their components are well conserved from yeast to humans, as well as in plants and algae (10,11). NPC’s structural components are known as nucleoporins (NUPs) and, even though the architecture of the NPCs is highly conserved, there exist significant differences in the NPC size and composition (12,13). Remarkably, unicellular eukaryotes appear to lack some NPC subunits (14,15). In general, the NPC is composed of four sub-compartments; a central transport channel, encompassed by a scaffold embedded in the nuclear envelope and two rings, one cytoplasmic and one nuclear, with eight filaments attached to each one. The nuclear ring filaments are connected to form a basket. Each NPC sub-complex is formed by different NUPs (Figure 1). About 34 different proteins characterized as nucleoporins exist (16). The NPC has an eightfold symmetry and as a result each NUP is present in multiples of eight. Nucleoporins are usually named based on their molecular mass, which may vary between orthologs, resulting in a slightly irregular nomenclature across different species (17).

While the endosymbiotic model adequately explains the evolutionary origins of plastids (18) the NPC origins remain unclear (19). Due to the high divergence of homologous NPC subunits at the sequence level, their deep evolutionary relations (as well as to other subunits of eukaryotic endomembrane systems) were proposed based on structural similarities (20,21), while only recently remote sequence similarity relations have been uncovered for the Y-complex nucleoporins and other eukaryotic endomembrane components (21). Additional evidence shows that the NPC can have different subunit composition in various species, while at the same time maintaining full functionality, a fact that also confirms the pore's plasticity (13,22,23). It is evident that increasing accumulation of numerous mutations in NPC subunits have resulted in viable proteins, which in turn developed new or similar functionalities that are not conserved across all eukaryotes (13,14); there are even cases where ‘lost’ nucleoporins of a lineage are compensated by neofunctionalization of other unrelated subunits (15). Moreover, nucleoporins have also drawn attention due to their involvement in unexpected functions (24,25), and in an array of diseases, such as age-related diseases (26), renal disorders (27–29), brain deficiencies (30,31), developmental conditions (32) and can even be the cause of heart attacks (33,34). Birth defects have also been accredited to mutations, particularly in the nucleoporin ALADIN, whose coding gene is named after the achalasia-adrenal failure, alacrima (absence of tears) syndrome (AAAS) (35,36).

The vast nucleoporin protein class contains a great number of orthologs, with some sharing family-specific functional domains which can be leveraged to facilitate the detection of newly characterized members, e.g. Nup160, NDC1 (37,38). However, there are observed cases, e.g. Nup37 (39), that do not contain unique characteristics to define a genuine family, but rather may contain common domains (e.g. WD40 and TPR repeats) that can also be found in proteins from unrelated families. This fact, in addition with the remarkable sequence divergence between orthologs and the presence of low complexity regions (LCRs) (24), turns the identification of non-annotated or newly sequenced nucleoporins into a major challenge.

Herein, we extend previous work (21,24) and characterize the diversity of NUP subunits across eukaryotes using multiple sequence alignment profiles. Based on curated sets of NUPs, we construct a library of position-specific scoring matrices (PSSMs) and further demonstrate their predictive value in detecting NUP subunits in proteins encoded in sequenced eukaryotic genomes. The NUP-PSSM library has wider coverage (with more NUP families represented) compared to NUP-specific profile Hidden Markov models currently available in PFAM (40). Furthermore, we show how PSSMs for fungal-specific NUP subunits can be used against a Metagenome Assembled Genome (MAG) dataset, serving as a diagnostic ‘signature’ for the origin of uncultured microbes. We believe that this comprehensive NUP-PSSM library can be a useful high-throughput sequence analysis tool for the effective and efficient in silico characterization of NUP subunits.

## MATERIALS AND METHODS

### Data collection

For the compilation of the initial protein sequence seed dataset, 36 protein sequences of the NPC were retrieved based on an authoritative review (41). For each NPC subunit, a single representative sequence was retrieved from the UniProtKB database (42). Where possible, *Drosophila melanogaster* proteins were selected; in case that a *Drosophila* protein was not available, *Homo sapiens* (e.g. POM121) or *Schizosaccharomyces pombe* (e.g. Mug31) protein sequences were used instead (Supplementary Table S1). These sequences were used as the base query to retrieve homologous nucleoporin protein sequences. It is noted that the choice of the reference query is not crucial at this point; *D. melanogaster* has been previously used as a starting point in nucleoporin protein data harvesting using sequence similarity techniques (24). A specific case is Nup96 and Nup98 which are encoded by a single gene, translated to a single polypeptide, and cleaved by its internal autopeptidase domain, commonly used to identify Nup96/98 orthologs; thus, for the aforementioned case only, a single sequence was retrieved. The final dataset of the 36 representative NPC subunit sequences was used to perform iterative BLAST searches to retrieve homologs from the NCBI non-redundant (nr) database.

### Nucleoporin family delineation: searching for homologs

Iterative sequence similarity searches using PSI-BLAST were executed for each NUP subunit type (24,43). PSI-BLAST runs reported herein were performed using the non-redundant protein database (nr) and the Entrez Query ‘NOT fragment NOT partial’, to exclude protein fragments or partial proteins and keep only complete sequences, avoiding low quality or ambiguous information. The parameter ‘max target sequences’ was set to 20 000 to retrieve the maximum number of results. The ‘Compositional adjustments’ parameter was set to ‘Composition based statistics’ – a setting observed to work well in combination with CAST masking (44) – as described in (24). CAST masking was executed with a modified threshold of 15. In order to avoid irrelevant hits, we further switched to a more stringent threshold and expected threshold values in a protein family-specific manner, based on the presence of repeats or compositionally biased regions (FG and TPR repeats, WD40 domains) in certain cases (Supplementary Table S2). Following a PSI-BLAST iteration, results were checked manually; in cases where a new hit was not annotated as a nucleoporin, reverse BLAST runs with the matching part of the sequence were used to ascertain detection. PSI-BLAST was terminated either when convergence was achieved or when the new results of an iteration started to retrieve unrelated hits. When all curated sequences for each NPC component were collected, PSI-BLAST was executed selectively for each family, to create sequence profiles, using the following parameter list: query = the family of unmasked sequences; db = the blast database containing sequences of the family; num_iterations = 0 (until convergence); max_hsps = 1 (1 hit per sequence) and out_pssm (export of the PSSM profile).

### Benchmarking the PSSMs

To test the accuracy of the resulting PSSM profiles, we have searched across several candidate UniProt reference proteomes from different taxonomic groups, including *Arabidopsis thaliana*, *Caenorhabditis elegans*, *Drosophila melanogaster*, *Danio rerio*, *Homo sapiens*, *Macaca mulatta*, *Mus musculus*, *Saccharomyces cerevisiae*, *Trypanosoma brucei* and *Xenopus laevis*. In addition to these well studied model species, we have also acquired the proteomes of species with experimentally studied Nuclear Pore Complex, namely *Chlamydomonas reinhardtii*, *Chaetomium thermophilum*, *Malus domestica*, *Schizosaccharomyces pombe* and *Tetrahymena thermophila* (Supplementary Table S3). We decided to not use less-well annotated genomes to evaluate the detection quality of the developed models. One reason for that, is the lack of quality functional annotations in these datasets that would serve as a golden standard against to which we would test our methods. In addition, several eukaryotic genomes may have substantial gaps and/or suffer from other quality issues (e.g. assembly errors, protein coding gene detection errors) which might affect any downstream analyses (45,46). The PSI-BLAST command line tool was used to query proteomes against the different PSSMs, using the following parameters: ‘CPU threads’ = 8, ‘number of iterations’ = 1, ‘composition-based statistics’ = 1, ‘max target sequences’ = 20 and ‘max hits per sequence’ = 1 (the latter two parameters chosen to reduce the output volume). Results were collected in BLAST tabular format and further processed; each of the hits was manually checked against UniProt to confirm that the NUP family represented by the matching PSSM corresponds to the true family of the respective protein. Finally, the same methodology was used with masked proteome sequences, using CAST (threshold: 15, all other parameters as default).

### Comparing against PFAM models

To evaluate detection sensitivity of PSSMs against existing methods, firstly we have selected the PFAM database (40) as the baseline, as it follows a similar principle of building family-specific profile Hidden Markov Models (HMMs – hereinafter PFAM-HMM) based on multiple sequence alignment characteristics. For this, the PFAM-A database was used, and the nucleoporin family models were identified and retrieved (where available; 26 families in total) (Supplementary Table S4). Using the *hmmsearch* (v.3.2.1) tool, part of the HMMER3 collection (47), we queried each proteome with the collected HMM profiles. Here, we have modified 2 parameters; the number of CPU threads (8), and the ‘max’ option which disables all heuristic filters, such as bias, increasing sensitivity. HMMER was also executed with heuristic filters enabled. Results in tabular format were further processed; each of the hits was manually checked against UniProt to confirm the protein family. Subsequently, the results were cross-referenced with the data from the PSSM-based detection described previously, thus allowing us to directly compare the two approaches. Finally, the same methodology was used with masked proteome sequences, using CAST (threshold: 15, all other parameters as default).

### Comparing against custom HMM models

In addition to the PFAM-HMM models, we have also created HMM models (hereinafter NUP-HMM) using the sequence alignments generated during the generation of PSSM profiles. For the detection of nucleoporins in the target proteomes, we have used the same methods and parameters as the previous step with the PFAM-HMM models, changing only the query HMM model. Each of the hits was manually checked against UniProt to ascertain the validity of protein family membership. Results were then cross-referenced with data from PSSM-based detection described previously, thus allowing us to directly compare the two methods. Finally, the same methodology was used with masked proteome sequences, using CAST (threshold: 15, all other parameters default).

## RESULTS

### Data harvesting: similarity searches

**Figure 2.**
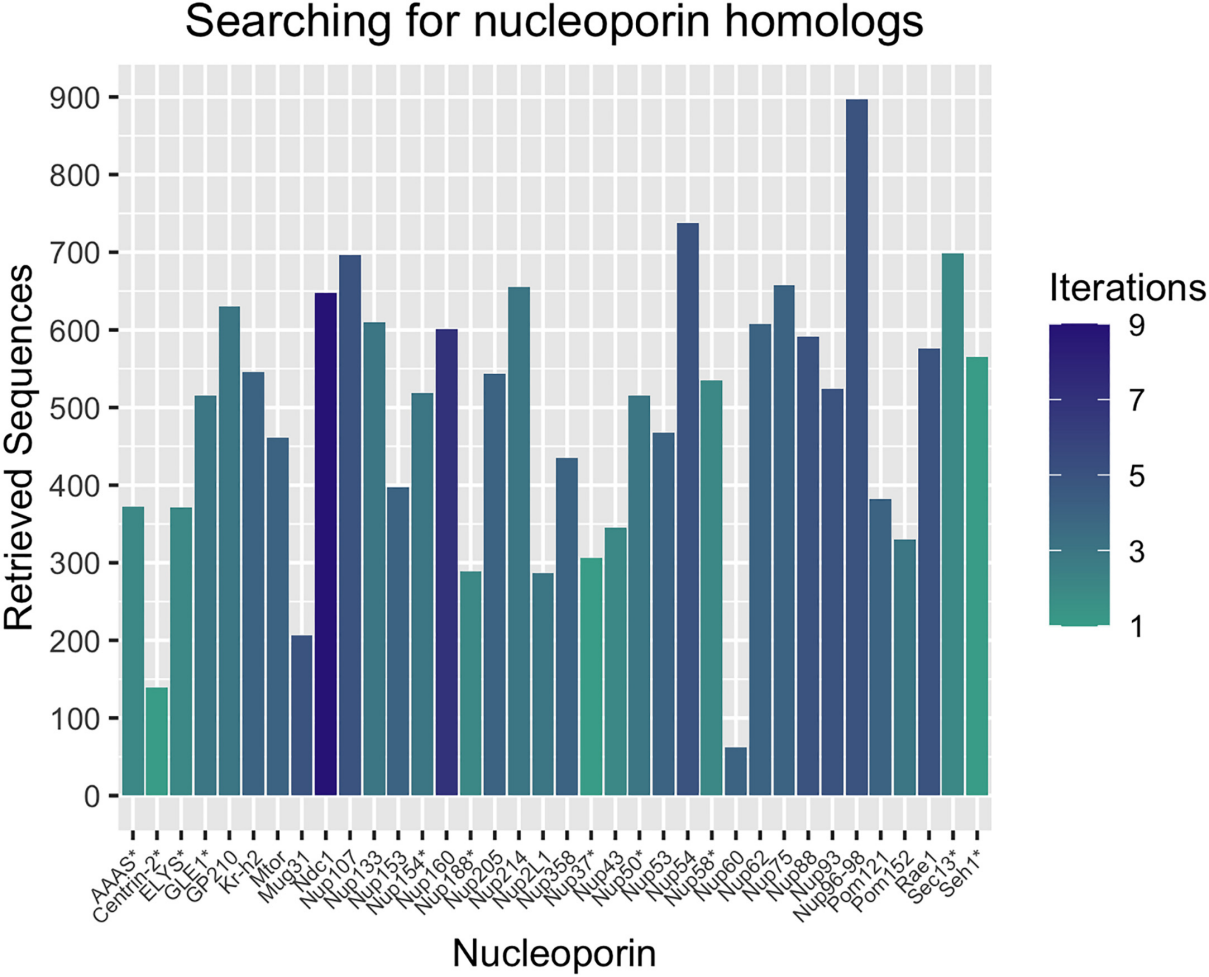
Homologous protein search statistics. Summary of sequences retrieved. Bar color coding based on the number of iterations during PSI-BLAST sequence similarity search. Nucleoporin families marked with asterisk were stopped before convergence.

The initial dataset consists of 36 protein sequences retrieved from UniProt (see Materials and Methods). During the iterative BLAST searches, we filtered any unrelated sequences (see Materials and Methods for details). After the final iteration, a total of 17 965 sequences were retrieved, of which 246 were removed since they were deemed as false positives, based on sequence domain composition or taxonomic distribution characteristics (Figure 2 and Supplementary Table S1). The majority of protein sequences identified as nucleoporins by the PSSM library are annotated in their description field as ‘hypothetical’ or ‘predicted’.

### Nucleoporin subunit detection and validation

Based on previous literature on the composition of NPCs across eukaryotes, we aimed to identify 406 proteins; this derives when subtracting 134 not expected to be found, from the theoretical maximum total of 540 NUP (= 15 × 36) subunits in 15 species. PSSMs of the NUP-PSSM library were able to detect 375 as top hits (Tables 1A, 2). The remaining 31 entries were not detected or ranked lower in the hit list. Based on these counts we obtain a true positive rate (TPR) of 92.4% and a false negative rate (FNR) of 7.6%.

**Table 1.** Coverage of detection of nucleoporins. (A) Detection using the constructed PSSM profiles (NUP-PSSM). (B) Detection using the available PFAM-HMM profiles, compared to the respective NUP-PSSM profiles (only applicable nucleoporin families, with existing PFAM-HMM profiles). (C) Detection using the constructed NUP-HMM profiles (from the PSI-BLAST alignments)

		PSSM		pHMM (no heuristics)		pHMM (heuristics)	
A. NUP-PSSM Library							
*n* = 406		Detected					
		Yes	No	Yes	No	Yes	No
Literature	Yes	375	31				
	No	0	134				
B. PFAM-HMM - PSSM library comparison							
*N* = 309		Detected					
		Yes	No	Yes	No	Yes	No
Literature	Yes	286	23	246	63	238	71
	No	0	81	0	81	0	81
C. NUP-HMM – PSSM library comparison							
*n* = 406		Detected					
		Yes	No	Yes	No	Yes	No
Literature	Yes	375	31	368	38	362	44
	No	0	134	0	134	0	134

**Table 2.** Position of the correct hit in the PSI-BLAST output on unmasked sequences. Red-colored boxes refer to proteins that are not considered detected

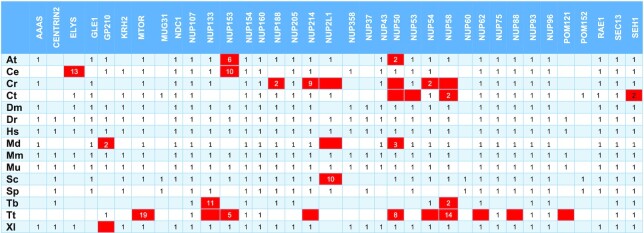

At – *Arabidopsis thaliana*, Ce – *Caenorhabditis elegans*, Cr – *Chlamydomonas reinhardtii*, Ct – *Chaetomium thermophilum*, Dm – *Drosophila melanogaster*, Dr – *Danio rerio*, Hs – *Homo sapiens*, Md – *Malus domestica*, Mm – *Macaca mulatta*, Mu – *Mus musculus*, Sc – *Saccharomyces cerevisiae*, Sp – *Schizosaccharomyces pombe*, Tb – *Trypanosoma brucei*, Tt – *Tetrahymena thermophila*, Xl – *Xenopus laevis*.

Regarding the detection using PFAM-HMMs with heuristic filters off, from a total of 309 proteins (theoretical maximum: 390) these models were able to detect 246 proteins (Tables 1B, 3A). Based on these counts, we obtain a TPR of 79.6% and a FNR of 20.4%. For the remaining 63 proteins, 25 were not detected at all and 38 ranked lower than their PSSM hit counterparts. Moreover, *Chlamydomonas reinhardtii* Nup188 and Nup58, and Nup50 from *Arabidopsis thaliana* and *Malus domestica*, ranked higher when queried with the PFAM-HMM profile and are the only occurrences of a PFAM-HMM profile hit ranking higher compared to a NUP-PSSM profile.

For the NUP-HMM library detection with heuristic filters off, from a total of 406 proteins (theoretical maximum: 540) these models were able to detect 368 as top hits (Tables 1C, 4A). As above, we obtain a TPR of 90.6% and a FNR of 9.4% in this case.

When both HMM profile libraries are queried with heuristic filters on (Tables 1B, C, 3B, 4B), it seems that the detection of some NPC subunits is not as effective. The PFAM-HMM library was able to detect as top hits 238 proteins out of 309 entries (TPR = 77.0%, FPR = 23.0%). In contrast, for the subset of common families, the respective PSSM figures are 92% TPR and 8% FNR, demonstrating that the NUP-PSSM library can be used to accurately delineate the composition of NPCs across eukaryotic species. For the NUP-HMM library, the results are better, compared to PFAM-HMM, as the models were able to detect 362 proteins from a total of 406, resulting in a TPR of 89.2% and a FNR of 10.8%, very close to what the NUP-PSSM library offers.

Regarding the exceptional case of *X. laevis* GP210 which is reported in the literature (40), yet non-detectable with any of the abovementioned profiles, it is worth mentioning that the respective sequence is not part of the *X. laevis* proteome in UniProt. UniProt/TrEMBL contains two unreviewed *X. laevis* entries annotated as GP210 (Q8JH73 and Q5IRN1); when these entries are appended to the *X. laevis* proteome they are correctly ranked at the top two positions using the GP210 profile from the NUP-PSSM and NUP-HMM libraries, and undetectable using the respective PFAM-HMM profiles.

Querying the datasets using both methods shows that the PSSM profiles provide more coverage of the nucleoporin superfamily and are also more sensitive as more proteins appear on the top of the hit list. Using the NUP-PSSM profiles, we observe a 12.8% and a 15.4% increase in detecting the correct protein as the first hit, in contrast to the PFAM-HMM profiles with heuristics filters off or on respectively. The respective increase when comparing the NUP-HMM library is 1.8% and 3.2% with heuristics filters off or on respectively. The detection rates of the NUP-HMM library, even if lower, are observed to be close to those of the NUP-PSSM library. These findings exemplify the value of using the NUP-PSSM profiles in high-throughput *in silico* detection pipelines, especially for classes as diverse as the nucleoporins.

Some nucleoporin instances are observed to rank lower in the hit lists, a fact that can be possibly attributed to several reasons, such as the existence of repeats and/or compositional biases in the sequences of particular NUP subunits. To investigate this issue, we followed the same methodology for proteomes masked with CAST (using default parameters), as masking is expected to eliminate spurious hits due to local compositional biases; since CAST does not use a window parameter, it is able to detect compositional biases spanning across complete sequences (44). We observe that reduces sensitivity for NUP-PSSM search, as 16 proteins rank lower than the 1^st^ position and 18 are not detected (Table 2, Supplementary Table S5). In the case of PFAM-HMM with heuristics off, 67 proteins are not detected with some ranks even lower that the unmasked counterparts (e.g. Centrin-2) (Table 3A, Supplementary Table S6). Nup214 was not detected in *H. sapiens, M. mulatta* and *M. musculus*. It seems that in some cases masking has improved detection, as Nup61 in *T. brucei*, Nup50 in *C. elegans* and Nup154-Nup358 in *D. melanogaster* now rank first. Finally, as expected, turning on the heuristic filters further reduces sensitivity and detection (Table 3B, Supplementary Table S7), as 77 proteins are not detected. The most obvious case being Centrin-2, which previously ranked higher (even with a generic EF-hand profile), now ranks lower than 65. In addition, a lot of members from Nup133, Nup153 and Nup214 families are not detected. As with unmasked sequences, the PSSM profiles outperform PFAM-HMMs (Supplementary Tables S5–S7). Finally, it is noted that the NUP-HMM library, also outperforms the PFAM-HMM library when queried against masked sequences (Supplementary Tables S6–S9). With heuristics off, 365 proteins were detected, while 41 were not. With heuristics on, 361 proteins were detected and 45 were not. These result in a TPR of 89.9% and 88.9% respectively.

### Performance evaluation

**Figure 3.**
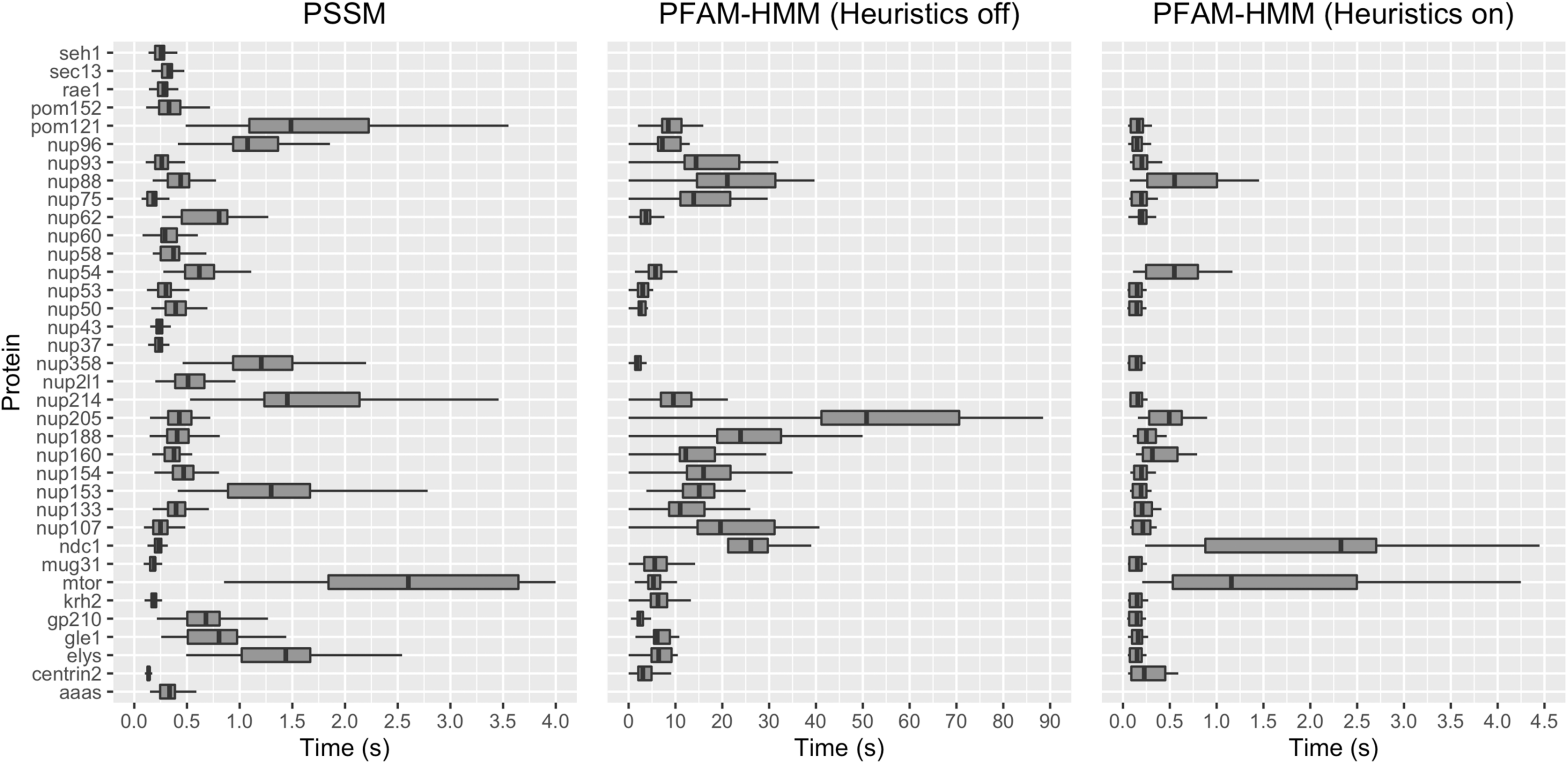
Time required to query each profile against proteomes. The execution times were measured 10 times. PFAM-HMM profiles are shown only where applicable. PSSM querying is completed faster in comparison to PFAM-HMM with heuristic filters off. When heuristics filters are enabled, execution times for PFAM-HMM are similar to PSSM querying times, and in some cases faster.

**Figure 4.**
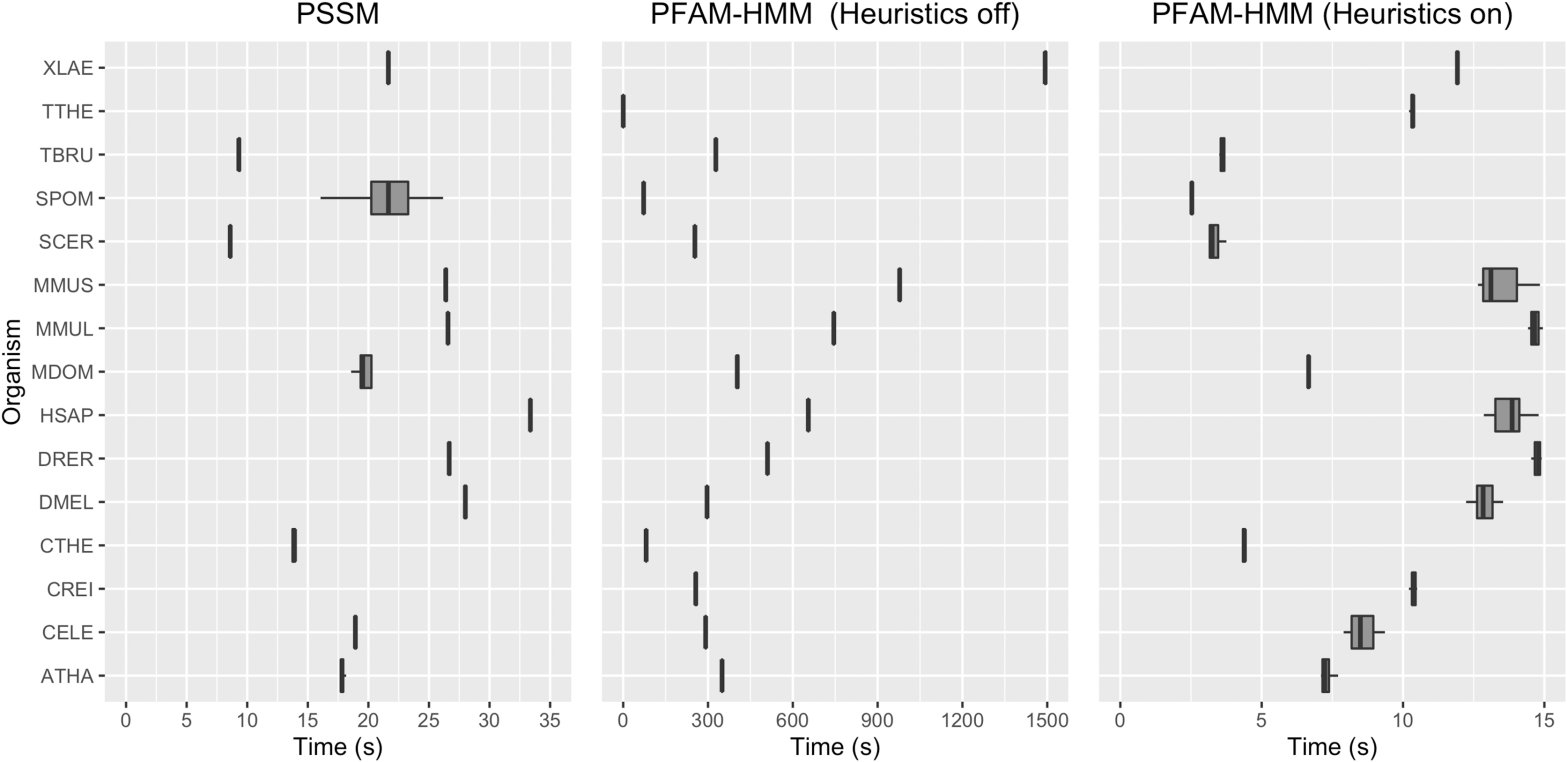
Time required to query the whole set of profiles against proteomes. The execution times were measured 10 times. PSSM querying is completed faster in comparison to PFAM-HMM with heuristic filters off. When heuristics filters are enabled, execution times for PFAM-HMM is noticeably faster than PSSM.

Execution times of all three methods (PSI-BLAST with PSSMs, HMMSEARCH with PFAM-HMMs and NUP-HMMs) were also calculated to obtain an indication for their applicability in high-throughput, automated proteome annotation settings. It should be noted that we tried to keep the run parameters of each tool (to the best extent) similar, such as the number of CPU threads. Execution time statistics were compiled both at the proteome level (processing of the whole proteome with the respective model) and at the individual protein family level. The metrics denoted here consist of the total and average time needed for: (a) each profile to query all proteomes (Figure 3); and (b) the whole set of profiles to query a single proteome (Figure 4). For both cases the queries were run 10 times and the runtime median was recorded.

The more sensitive PFAM-HMM mode (heuristic filters off) requires more time to execute compared to the NUP-PSSM models. When the heuristic filters are on, PFAM-HMM models perform on average in par to their PSSM counterparts, however with much less sensitivity (15.4% reduction in TPR). Therefore, the NUP-PSSM library can provide much higher NUP sensitivity with comparable computational performance to PFAM hmmsearch queries. Looking at the same statistics for the NUP-HMM library, it is observed that this method performs worse than all other methods, an event attributed to the size of the alignments used to create the NUP-HMM profiles (Supplementary Figures S1 and S2). Finally, it is observed that to some extent, the execution time is relative to the query profile length (Supplementary Figure S3). Median time of execution does not present large deviations when looking at the HMM profiles with heuristics off, while all other cases present larger deviations from the trend. An analysis was performed on whether the low complexity regions (LCRs) are involved in these deviations (test case was the PSSM profiles), which has been inconclusive, showing no direct association of LCR content with execution time (data available on GitHub repository).

### Case study: unveiling fungal metagenome-assembled genomes

Metagenomes are constructed by sequencing of all genomic material in an environmental sample (48). Recent advances in metagenomic sequencing and metagenome assembly pipelines have enabled the reconstitution of metagenome-assembled genomes (MAGs), which can be thought of as assemblies of hypothetical free-living species, mined from metagenomic data. A recent study on the reduction of redundancy of single cell eukaryotic MAGs (49) identified 99 073 prokaryotic and 751 eukaryotic genomes. Within this dataset of eukaryotic MAGs (ENA Accession Number PRJEB51083), the authors characterized a subset as eukaryotic species representatives (*n* = 124) based on Aligned Fraction and Average Nucleotide identity to isolate genomes. These MAGs were assigned to fungi (98 MAGs), algae (19 MAGs) and protozoa (7 MAGs). Since the NUP-PSSM library contains 3 fungi-specific models (i.e. for Pom152, Mug31 and Nup60), we set to investigate whether these models are sensitive enough to detect the respective fungal nucleoporins in these metagenomic-derived data. In addition, such an analysis serves as a benchmark for the specificity of these models: for example, Nup60 which is known to possess FG-repeats (9) might produce false positives with other FG nucleoporins from non-fungal species in this dataset. Data were provided without a taxonomic assignment, thus serving as a blind test to validate detection of NUPs in fungi. A total of 1 065 617 protein sequences were analyzed: initially, sequences were masked for compositionally biased regions using CAST (threshold: 15, all other parameters as default) and subsequently were scanned using the PSSMs representing the fungal-specific nucleoporin families, namely Pom152, Mug31 and Nup60. For comparison, runs with unmasked protein sequences were also performed with the above-mentioned PSSMs. In addition, we validated the only currently available PFAM model describing the Mug31 nucleoporin family (i.e. PF08058.8 - NPCC) run both with heuristics ‘off’ or ‘on’, and with or without sequence masking for compositional bias.

Interestingly, when screening the protein sequences encoded in the representative eukaryotic MAGs, a total of 98 MAGs returned at least one significant hit to one of the fungal-specific nucleoporin PSSMs (Table 5). The authors of this dataset confirmed that 97 out of the 98 MAGs with a fungal-specific PSSM hit were assigned to fungal species as well according to their independent analyses. A single false negative case is that of a MAG initially classified as *Malassezia sympodialis*, which had no detectable hits by our approach. Additionally, one false positive hit was identified, namely a putative Nup62/NSP1 nucleoporin from a *Blastocysis sp subtype 1* MAG. For this particular MAG, we also applied the Nup62 PSSM, which was detected as the top hit. Comparing the Nup60 and Nup62 hits on the MAG we observe the same detected region, due to the presence of the FG repeats. Finally, it is observed that masking sequences limits the number of hits, and both PSSMs and PFAM-HMMs tend to be more sensitive, although even in this case the PSSM outperforms the PFAM-HMM which returns a lot of false positive instances (Supplementary Table S10).

## DISCUSSION

The nuclear pore complex is present in all eukaryotic species, exhibiting substantial variance in its composition along the evolutionary trajectory. Even though the NPC and its subcomponents are well studied, not many high throughput sensitive methods are available for detecting their presence in proteomes. Here, we have focused on the delineation of nucleoporin families based on publicly available data and tools and established family-specific PSSM profiles for each nucleoporin protein family that can be readily deployed for the detection of the NPC components in eukaryotes.

We have performed extensive protein database queries to extract a high-quality curated repository of nearly 18000 nucleoporin sequences. Using the harvested information, we have constructed a PSSM family profile for each of the 36 different nucleoporin families. Subsequently, we have put these profiles to test by querying the proteomes of 15 eukaryotic species. This analysis was also compared with existing detection methods, and specifically with one set of relevant profile HMMs that were retrieved from PFAM, and another set of HMMs constructed from sequence alignments of the collected proteins. Our results indicate that PSSM profiles are more effective in identifying the correct sequence in the genome with high sensitivity, than both PFAM-HMMs and NUP-HMMs, with virtually having a result at first rank of the searches. In addition, PFAM-HMMs have shown a decrease in sensitivity for certain families, such as Centrin-2, Nup133, Nup154 and Nup214. For the cases of Nup133 and Nup154, PFAM-HMMs seem to be detecting mostly the other family, while PSSM and NUP-HMM profiles do not seem to be presenting this behavior. The reason for these observations emanates from HMM profiles, which correspond to non-specific domains, also found in other proteins. Specifically, the selected EF-Hand 7 profile of Centrin-2 is found in a wide range of calcium-binding proteins, e.g. Calmodulin-1 and Troponin C that often rank higher than Centrin-2. For Nup133 and Nup154, Nucleoporin_N and Nucleoporin_C HMM profiles respectively can be found in the N- and C- termini of such nucleoporins; again, this causes the query to cross-detect another family. Thus, for some families, PFAM-HMM profiles do not offer specificity and the only option is to select a sub-optimal motif to attempt detection. This behavior is not observed for PSSM or NUP-HMM profiles, as these are curated and constructed to provide both specific and sensitive family-specific instances. If Centrin-2, Nup133 and Nup154 families are excluded, then PFAM-HMM profiles present a TPR of 86% and 85% for heuristic filters off and on respectively, while the PSSM TPR remains at 92%. When taking into consideration the above exceptions NUP-HMM profiles present a TPR or 90% and 89.7% for heuristic filters off and on respectively. Moreover, we were also able to test all three methods with *T. brucei*. NUP-PSSMs have been able to identify all literature reported proteins, with most scoring the correct hit first (Table 2), whereas in some of the cases (Tables 3A, 3B) PFAM-HMMs rank lower. The NUP-HMM profiles better detect *T. brucei* nucleoporins when compared to PFAM models, with their detection rate being almost similar to those of NUP-PSSM (Table 4A, 4B); they even detect Nup133 as the top hit.

**Table 3A.** Position of the correct hit in the HMMSEARCH output with heuristic filters off and unmasked sequences, using PFAM-HMM profiles. Grey highlighted cells denote that the corresponding family PFAM-HMM profile does not exist. Red-colored boxes refer to proteins that are not considered detected

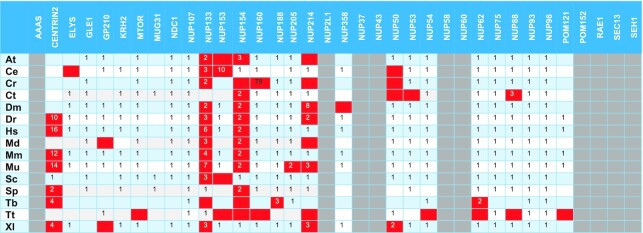

At – *Arabidopsis thaliana*, Ce – *Caenorhabditis elegans*, Cr – *Chlamydomonas reinhardtii*, Ct – *Chaetomium thermophilum*, Dm – *Drosophila melanogaster*, Dr – *Danio rerio*, Hs – *Homo sapiens*, Md – *Malus domestica*, Mm – *Macaca mulatta*, Mu – *Mus musculus*, Sc – *Saccharomyces cerevisiae*, Sp – *Schizosaccharomyces pombe*, Tb – *Trypanosoma brucei*, Tt – *Tetrahymena thermophila*, Xl – *Xenopus laevis*.

**Table 3B.** Position of the correct hit in the HMMSEARCH output with heuristic filters and unmasked sequences, using PFAM-HMM profiles. Grey highlighted cells denote that the corresponding family PFAM-HMM profile does not exist. Red-colored boxes refer to proteins that are not considered detected

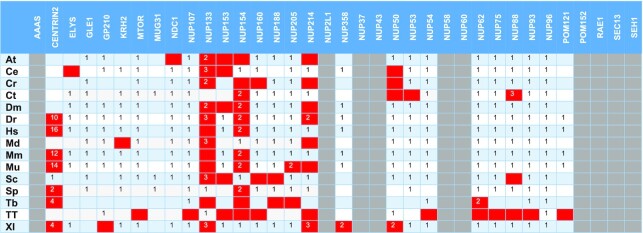

At – *Arabidopsis thaliana*, Ce – *Caenorhabditis elegans*, Cr – *Chlamydomonas reinhardtii*, Ct – *Chaetomium thermophilum*, Dm – *Drosophila melanogaster*, Dr – *Danio rerio*, Hs – *Homo sapiens*, Md – *Malus domestica*, Mm – *Macaca mulatta*, Mu – *Mus musculus*, Sc – *Saccharomyces cerevisiae*, Sp – *Schizosaccharomyces pombe*, Tb – *Trypanosoma brucei*, Tt – *Tetrahymena thermophila*, Xl – *Xenopus laevis*.

**Table 4A.** Position of the correct hit in the HMMSEARCH output with heuristic filters off and unmasked sequences, using custom HMM profiles (NUP-HMM) based on collected sequences. Red-colored boxes refer to proteins that are not considered detected

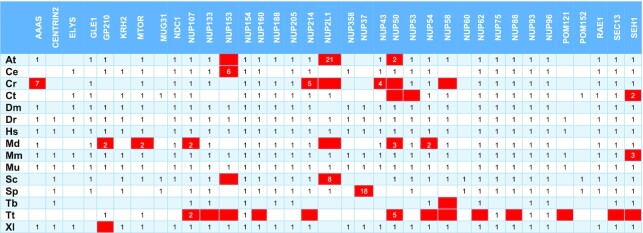

At – *Arabidopsis thaliana*, Ce – *Caenorhabditis elegans*, Cr – *Chlamydomonas reinhardtii*, Ct – *Chaetomium thermophilum*, Dm – *Drosophila melanogaster*, Dr – *Danio rerio*, Hs – *Homo sapiens*, Md – *Malus domestica*, Mm – *Macaca mulatta*, Mu – *Mus musculus*, Sc – *Saccharomyces cerevisiae*, Sp – *Schizosaccharomyces pombe*, Tb – *Trypanosoma brucei*, Tt – *Tetrahymena thermophila*, Xl – *Xenopus laevis*.

**Table 4B.** Position of the correct hit in the HMMSEARCH output with heuristic filters and unmasked sequences, using custom HMM profiles (NUP-HMM) based on collected sequences. Red-colored boxes refer to proteins that are not considered detected

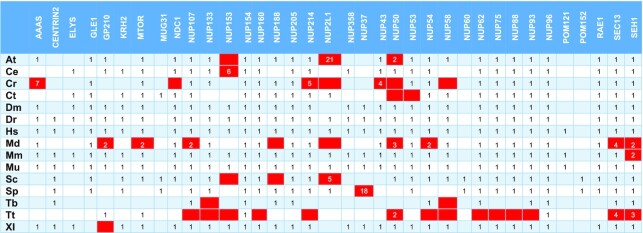

At – *Arabidopsis thaliana*, Ce – *Caenorhabditis elegans*, Cr – *Chlamydomonas reinhardtii*, Ct – *Chaetomium thermophilum*, Dm – *Drosophila melanogaster*, Dr – *Danio rerio*, Hs – *Homo sapiens*, Md – *Malus domestica*, Mm – *Macaca mulatta*, Mu – *Mus musculus*, Sc – *Saccharomyces cerevisiae*, Sp – *Schizosaccharomyces pombe*, Tb – *Trypanosoma brucei*, Tt – *Tetrahymena thermophila*, Xl – *Xenopus laevis*.

**Table 5.** Coverage of MAG detection using PSSM and PFAM-HMM profiles

		PSSM		PFAM-HMM (heuristics off)		PFAM-HMM (heuristics on)	
*n* = 124	Identified as fungal	Masked	Unmasked	Masked	Unmasked	Masked	Unmasked
Fungal (*n* = 98)	Y	97	98	89	98	70	78
	N	1	–	9	–	28	21
Non-fungal (*n* = 26)	Y	1	20	17	26	–	2
	N	25	6	9	–	26	23

It should be noted that even if a particular nucleoporin was not expected to be found in some taxa, e.g. the fungal-specific Nup60, Mug31 and POM152, we still exhaustively queried non-fungal species with those PSSM profiles, to understand how various proteomes score against profiles and establish family-specific exclusion thresholds to reduce noise. This is a framework for future experiments to assess sensitivity even for genomes with high content of low-complexity regions (9,50), such as trypanosomes or *Plasmodium* species.

We have tested the performance of the approach, concluding that protein detection using PSSM profiles is more efficient than HMM profiles at comparable sensitivity, completing full proteome scans in seconds. Generally, detection methods perform better when querying unmasked proteomes, more so for PSSM profiles and to a lesser extent for PFAM-HMMs. Using masked sequences results in a slight decrease of sensitivity in PSSM profiles. PFAM-HMMs also lose sensitivity and coverage, but this seems to be restricted to a few NPC subunits, such as Centrin-2, Nup133, Nup153, Nup154 and Nup214. This is not the case for NUP-HMM profiles, as they outperform their PFAM counterparts, with higher detection rates regardless of the configuration (heuristics on/off) or sequence masking.

It is interesting to further investigate whether the detection performance of the NUP-PSSM and NUP-HMM profiles is correlated with the properties of the input multiple sequence alignments (e.g. number of sequences and alignment length). When a profile-HMM is trained on a multiple sequence alignment using HMMER, particular columns are designated as insertion or deletion states; this is a powerful feature of profile-HMMs which (in addition to the remaining ‘match’ states) enables them to model gaps more effectively compared to simple profiles, e.g. PSSMs. In our data even though trained with the same input multiple alignments, the lengths of the constructed NUP-HMMs often deviate considerably (>5%) from the lengths of the respective NUP-PSSM models. We observe that when this deviation is higher, PSSMs more frequently overperform the respective profile-HMMs (Supplementary Table S11). A similar trend (although weaker) is observed when model sensitivity is compared against the depth of the multiple alignment, using as a proxy the effective number of sequences (Supplementary Table S11). However, the data sample on which these observations are based (15 proteomes) is relatively small to make definitive conclusions.

Finally, a blind experiment carried out to test fungi detection performance against unannotated metagenome-derived data has been instructive. The fungal-specific subset of the NUP-PSSMs was used to detect 97/98 MAGs (out of a set of 124 MAGs of eukaryotic origin) as hypothetical fungal species. One undetected fungal MAG needs to be further investigated (e.g. in terms of genome completeness), while the single false positive result was due to a false detection of another FG-repeat containing nucleoporin (not shown). The undetected PSSM hit may be attributed to the protein missing from the data as MAGs can be partially complete.

To conclude, we report a repository of nucleoporin protein sequences and associated sensitive family-specific PSSM profiles. These profiles can be used in high-throughput proteome analysis to detect the presence of nucleoporins in various organisms with sensitivity and specificity. The PSSM profiles developed for the purposes of this study are made available (see Data Availability) and may be used to further our understanding on the structure, function, and evolution of the nuclear pore complex, especially for newly sequenced eukaryotic species.

## DATA AVAILABILITY

Nucleoporin sequences, profiles and results presented in this paper available at: https://doi.org/10.5281/zenodo.7684166.

## Supplementary Material

lqad025_Supplemental_File
