# Supplementary material for: A library of sensitive position-specific scoring matrices for high-throughput identification of nuclear pore complex subunits
Source: NAR Genom Bioinform. 2023 Mar 23;5(1):lqad025. doi: 10.1093/nargab/lqad025 (PMC10034585; doi:10.1093/nargab/lqad025)
Supplement: lqad025_Supplemental_File [file lqad025_supplemental_file.pdf]

Supplementary Table 1. Proteins retrieved from UniProt. Nup96-98 have the same accession number as they are encoded by a single gene. PSI-BLAST iterations marked with asterisk denote that the search has not converged.

NPC Component	Protein Name	Organism	Uniprot Accession Number	Sequences Retrieved	PSI-BLAST Iterations
Cytoplasmic filaments	Nup358	*D. melanogaster*	Q9VBU7	435	4
Cytoplasmic ring and associated factors	Nup214	*D. melanogaster*	Q9W1X4	655	3
	Nup88	*D. melanogaster*	Q9GYU8	591	5
	GLE1	*D. melanogaster*	Q9V4W1	515	3*
	Nup2L1	*H. sapiens*	O15504	287	3
Nup98 complex	Nup98	*D. melanogaster*	Q867W7	In single gene with Nup96	
	Rae1	*D. melanogaster*	Q9W2E7	576	5
Outer NPC scaffold	Nup160	*D. melanogaster*	Q9VKJ3	601	7
	Nup133	*D. melanogaster*	Q9VCW3	610	3
	Nup107	*D. melanogaster*	A1YK54	696	5
	Nup96	*D. melanogaster*	Q867W7	897	5
	Nup75	*D. melanogaster*	A1YK02	657	4
	Seh1	*D. melanogaster*	Q7K2X8	565	1*
	Sec13	*D. melanogaster*	Q9V3J4	699	2*
	Nup37	*D. melanogaster*	Q9VBU8	306	1*
	Nup43	*D. melanogaster*	Q9VE85	345	2
	Centrin-2	*H. sapiens*	P41208	139	1*
Central NPC scaffold	Nup205	*D. melanogaster*	Q8IQV9	544	4
	Nup188	*H. sapiens*	Q5SRE5	289	2*
	Nup154	*D. melanogaster*	Q9V463	519	3*
	Nup93	*D. melanogaster*	Q9VFE7	524	5
	Nup53	*D. melanogaster*	Q9VWS2	468	4
Nup62 complex	Nup62	*D. melanogaster*	Q7JXF5	608	4
	Nup58	*D. melanogaster*	Q9VDV3	535	2*
	Nup54	*D. melanogaster*	Q9V6B9	738	5
Nuclear ring and associated factors	Nup153	*D. melanogaster*	Q9VXE6	397	4
	Nup50	*D. melanogaster*	Q7K0D8	515	3*
	Nup60	*S. pombe*	O74500	62	4
Nuclear basket	Mtor	*D. melanogaster*	A1Z8P9	461	4
Transmembrane nucleoporins	Ndc1	*D. melanogaster*	Q9VCG4	648	9
	Pom12	*H. sapiens*	Q96HA1	382	4
	GP210	*D. melanogaster*	A1Z6H7	630	3
	Kr-h2	*D. melanogaster*	Q9V447	546	4
	Pom152	*S. pombe*	O94385	330	3
	Mug31	*S. pombe*	Q9US56	206	5
Other	ELYS	*H. sapiens*	Q8WYP5	371	2*
	AAAS	*H. sapiens*	Q9NRG9	372	2*

Supplementary Table 2. Changes in PSI-BLAST threshold values for each family.

NPC component	Protein Name	PSI-BLAST Threshold	Expected Threshold
Cytoplasmic filaments	Nup358	1e-4	1e-6
Cytoplasmic ring and associated factors	Nup214	1e-4	1e-6
	Nup88	1e-4	10 (def.)
	GLE1	1e-4	10 (def.)
	Nup2L1	1e-4	10 (def.)
Nup98 complex	Nup98	5e-3 (def.)	10 (def.)
	Rae1	1e-4	1e-6
Outer NPC scaffold	Nup160	5e-3 (def.)	10 (def.)
	Nup133	5e-3 (def.)	10 (def.)
	Nup107	5e-3 (def.)	10 (def.)
	Nup96	5e-3 (def.)	10 (def.)
	Nup75	5e-3 (def.)	10 (def.)
	Seh1	1e-4	1e-6
	Sec13	1e-4	1e-6
	Nup37	1e-4	1e-6
	Nup43	5e-3 (def.)	10 (def.)
	Centrin-2	5e-3 (def.)	10 (def.)
Central NPC scaffold	Nup205	5e-3 (def.)	10 (def.)
	Nup188	5e-3 (def.)	10 (def.)
	Nup154	5e-3 (def.)	10 (def.)
	Nup93	5e-3 (def.)	10 (def.)
	Nup53	5e-3 (def.)	10 (def.)
Nup62 complex	Nup62	1e-4	10 (def.)
	Nup58	1e-4	10 (def.)
	Nup54	1e-4	10 (def.)
Nuclear ring and associated factors	Nup153	1e-4	1e-6
	Nup50	1e-4	1e-6
	Nup60	1e-4	1e-6
Nuclear basket	Mtor	1e-4	1e-6
Transmembrane nucleoporins	Ndc1	5e-3 (def.)	10 (def.)
	Pom12	5e-3 (def.)	10 (def.)
	GP210	1e-4	10 (def.)
	Kr-h2	1e-4	10 (def.)
	Pom152	5e-3 (def.)	10 (def.)
	Mug31	5e-3 (def.)	10 (def.)
Other	ELYS	1e-4	10 (def.)
	AAAS	1e-4	1e-6

Supplementary Table 3. The UniProt proteome IDs.

Organism	UniProt Proteome ID
Arabidopsis thaliana	UP000006548
Caenorhabditis elegans	UP000001940
Chlamydomonas reinhardtii	UP000006906
Chaetomium thermophilum	UP000008066
Drosophila melanogaster	UP000000803
Danio rerio	UP000000437
Homo sapiens	UP000005640
Malus domestica	UP000290289
Macaca mulatta	UP000006718
Mus musculus	UP000000589
Saccharomyces cerevisiae	UP000002311
Schizosaccharomyces pombe	UP000002485
Trypanosoma brucei	UP000008524
Tetrahymena thermophila	UP000009168
Xenopus laevis	UP000186698

Supplementary Table 4. PFAM HMM ID mapping with nucleoporin families.

Nucleoporin Family	PFAM HMM ID	PFAM Description
Centrin2	PF13499.3	EF-hand_7
Elys	PF13934.3	ELYS
GLE1	PF07817.10	GLE1
Krh2	PF03661.10	TMEM33_Pom33
Mtor	PF07926.9	TPR_MLP1_2
Mug31	PF08058.8	NPCC
Ndc1	PF09531.7	Ndc1_Nup
Nup50	PF08911.8	NUP50
Nup53	PF05172.10	Nup35_RRM
Nup54	PF13874.3	Nup54
Nup62	PF05064.10	Nsp1_C
Nup75	PF07575.10	Nucleopor_Nup85
Nup88	PF10168.6	Nup88
Nup214	PF16755.2	NUP214
Nup93	PF04097.11	Nic96
Nup96	PF12110.5	Nup96
Nup107	PF04121.10	Nup84_Nup100
Nup133	PF08801.8	Nucleoporin_N
Nup153	PF08604.7	Nup153
Nup154	PF03177.11	Nucleoporin_C
Nup188	PF10487.6	Nup188
Nup205	PF11894.5	Nup192
Nup358	PF00638	Ran_BP1
Pom121	PF15229.3	POM121
Nup160	PF11715.5	Nup160
GP210	PF02368.15	Big_2
Nup43	N/A	

Nup58	N/A
Seh1	N/A
Pom152	N/A
Nup2L1	N/A
Nup37	N/A
AAAS	N/A
Sec13	N/A
Nup60	N/A
RAE1	N/A

Supplementary Table 5. Position of the correct hit in the PSI-BLAST output on masked sequences. Red-colored boxes refer to proteins that are not considered detected.

	AAAS	CENTRIN2	ELYS	GLE1	GP210	KRH2	MTOR	MUG31	NDC1	NUP107	NUP133	NUP153	NUP154	NUP160	NUP188	NUP205	NUP214	NUP2L1	NUP358	NUP37	NUP43	NUP50	NUP53	NUP54	NUP58	NUP60	NUP62	NUP75	NUP88	NUP93	NUP96	POM121	POM152	RAE1	SEC13	SEH1
**At**	1			1	1		1		1	1	1	5	1	1	1	1	1	1			1	2	1	1	1		1	1	1	1	1			1	1	1
**Ce**			1		1	1	1		1	1	1	3	1	1		1	1		1		1	1	1	1			1	1	1	1	1			1	1	1
**Cr**	9			1			1		1	1	1		1	1		1					2		1	1	1		1	1	1	1	1			1	1	1
**Ct**			1	1		1	1	1	1	1			1	1	1	1	1	1						1	1		1	1	1	1	1		1	1	1	2
**Dm**	1		1	1	1	1	1		1	1	1	1	1	1	1	1	1		1	1	1	1	1	1	1		1	1	1	1	1			1	1	1
**Dr**	1	1	1	1	1	1	1		1	1	1	1	1	1	1	1	1	1	1	1	1	1	1	1	1		1	1	1	1	1	1		1	1	1
**Hs**	1	2	1	1	1	1	1		1	1	1	1	1	1	1	1	1	1	1	1	1	1	1	1	1		1	1	1	1	1	1		1	1	1
**Md**	1			1	2		1		1	1	1		1	1	1	1	1				1	3	1	2	1		1	1	1	1	1			1	1	1
**Mm**	1	1	1	1	1	1	1		1	1	1	1	1	1	1	1	1	1	1	1	1	1	1	1	1		1	1	1	1	1	1		1	1	1
**Mu**	1	1	1	1	1	1	1		1	1	1	1	1	1	1	1	1	1	1	1	1	1	1	1	1		1	1	1	1	1	1		1	1	1
**Sc**		1		1		1	1	1	1	1	1		1	1	1	1	1	10				1	1	1		1	1	1	1	1	1		1	1	1	1
**Sp**		1		1		1		1		1	1		1	1	1	1	1	1		2			1			1	1	1	1	1	1		1	1	1	1
**Tb**		1								1			1		1	1								1			1			1	1				1	1
**Tt**					1		19			1		5	1	1								8			14			1		2	1				1	1
**Xl**	1	1	1			1	1		1	1	1	1	1	1	1	1	1		1	1	1	1	1	1	1		1	1	1	1	1			1	1	1

Legend: At – Arabidopsis thaliana, Ce – Caenorhabditis elegans, Cr - Chlamydomonas reinhardtii, Ct - Chaetomium thermophilum, Dm – Drosophila melanogaster, Dr – Danio rerio, Hs – Homo sapiens, Md - Malus domestica, Mm – Macaca mulatta, Mu – Mus musculus, Sc – Saccharomyces cerevisiae, Sp - Schizosaccharomyces pombe, Tb – Trypanosoma brucei, Tt - Tetrahymena thermophila , Xl – Xenopus laevis

Supplementary Table 6. Position of the correct hit in the HMMSEARCH output with heuristic filters off and masked sequences, using PFAM-HMM profiles. Grey highlighted cells denote that the corresponding family HMM profile does not exist. Red-colored boxes refer to proteins that are not considered detected.

	AAAS	CENTRIN2	ELYS	GLE1	GP210	KRH2	MTOR	MUG31	NDC1	NUP107	NUP133	NUP153	NUP154	NUP160	NUP188	NUP205	NUP214	NUP2L1	NUP358	NUP37	NUP43	NUP50	NUP53	NUP54	NUP58	NUP60	NUP62	NUP75	NUP88	NUP93	NUP96	POM121	POM152	RAE1	SEC13	SEH1
**At**				1	1		1		1	1	2		3	1	1	1						1	1	1			1	1	1	1	1					
**Ce**					1	1	1		1	1	8	10	1	1		1	1		1			1	1	1			1	1	1	1	1					
**Cr**				1					1	1	2					1							1	1			1	1	1	1	1					
**Ct**			1	1		1	1	1	1	1			2	1	1	1	1						1	1			1	1	1	1	1					
**Dm**			1	1	1	1	1		1	1	2		1	1	1	1	8		1			1	1	1			1	1	1	1	1					
**Dr**		10	1	1	1	1	1		1	1	3	1	2	1	1	1	2		1			1	1	1			1	1	1	1	1	1				
**Hs**		16	1	1	1	1	1		1	1	6	1	2	1	1	1			1			1	1	1			1	1	1	1	1	1				
**Md**				1			1		2	1	3		4	2	1	1						1	1	1			1	1	1	1	1					
**Mm**		12	1	1	1	1	1		1	1	4	1	2	1	1	1			1			1	1	1			1	1	1	1	1	1				
**Mu**		14	1	1	1	1	1		1	1	7	1	2	1	1	2			1			1	1	1			1	1	1	1	1	1				
**Sc**		2		1		1	1	1	1	1	3		1	1	1	1	1					1	1	1			1	1	1	1	1					
**Sp**		4		1		1		1		1	3		2	1	1	1	1						1				1	1	1	1	1					
**Tb**		4								1					2	1								1			1			1	1					
**Tt**							3			1				86																1	1					
**Xl**		4	1			1	1		1	1	3	1	3	1	1	1	3		1			2	1	1			1	1	1	1	1					

Legend: At – Arabidopsis thaliana, Ce – Caenorhabditis elegans, Cr - Chlamydomonas reinhardtii, Ct - Chaetomium thermophilum, Dm – Drosophila melanogaster, Dr – Danio rerio, Hs – Homo sapiens, Md - Malus domestica, Mm – Macaca mulatta, Mu – Mus musculus, Sc – Saccharomyces cerevisiae, Sp - Schizosaccharomyces pombe, Tb – Trypanosoma brucei, Tt - Tetrahymena thermophila , Xl – Xenopus laevis

Supplementary Table 7. Position of the correct hit in the HMMSEARCH output with heuristic filters on and masked sequences, using PFAM-HMM profiles. Grey highlighted cells denote that the corresponding family HMM profile does not exist. Red-colored boxes refer to proteins that are not considered detected.

	AAAS	CENTRIN2	ELYS	GLE1	GP210	KRH2	MTOR	MUG31	NDC1	NUP107	NUP133	NUP153	NUP154	NUP160	NUP188	NUP205	NUP214	NUP2L1	NUP358	NUP37	NUP43	NUP50	NUP53	NUP54	NUP58	NUP60	NUP62	NUP75	NUP88	NUP93	NUP96	POM121	POM152	RAE1	SEC13	SEH1
**At**				1	1		1			1	5			1	1	1						1	1	1			1	1	1	1	1					
**Ce**					1	1	1		1	1	3		1	1		1	1		1				1	1			1	1	1	1	1					
**Cr**				1					1	1	2					1							1	1			1	1	1	1	1					
**Ct**			1	1		1	1	1	1	1			2	1	1	1	1						1	1			1	1		1	1					
**Dm**			1	1		1	1		1	1	2		1	1	1	1			1			1	1	1			1	1	1	1	1					
**Dr**		88	1	1	1	1	1		1	1		1	2	1	1	1			1			1	1	1			1	1	1	1	1	1				
**Hs**		80	1	1	1	1	1		1	1		1	2	1	1	1			1			1	1	1			1	1	1	1	1	1				
**Md**				1			1		1	1	3			2	1	1						1	1	1			1	1	1	1	1					
**Mm**		69	1	1	1	1	1		1	1		1	2	1	1	1			1			1	1	1			1	1	1	1	1	1				
**Mu**		67	1	1	1	1	1		1	1		1	2	1	1	2			1			1	1	1			1	1	1	1	1	1				
**Sc**		2		1		1	1	1	1	1	3		1		1	1	1					1	1	1			1	1		1	1					
**Sp**		4		1		1		1		1	3		2	1	1	1	1						1				1	1	1	1	1					
**Tb**		12								1														1			1			1	1					
**Tt**																															1					
**Xl**		4	1			1	1		1	1	3	1	1	1	1	1	3		2			2	1	1			1	1	1	1	1					

Legend: At – Arabidopsis thaliana, Ce – Caenorhabditis elegans, Cr - Chlamydomonas reinhardtii, Ct - Chaetomium thermophilum, Dm – Drosophila melanogaster, Dr – Danio rerio, Hs – Homo sapiens, Md - Malus domestica, Mm – Macaca mulatta, Mu – Mus musculus, Sc – Saccharomyces cerevisiae, Sp - Schizosaccharomyces pombe, Tb – Trypanosoma brucei, Tt - Tetrahymena thermophila , Xl – Xenopus laevis

Supplementary Table 8. Position of the correct hit in the HMMSEARCH output with heuristic filters off and masked sequences, using custom HMM profiles (NUP-HMM) based on collected sequences. Red-colored boxes refer to proteins that are not considered detected.

	AAAS	CENTRIN2	ELYS	GLE1	GP210	KRH2	MTOR	MUG31	NDC1	NUP107	NUP133	NUP153	NUP154	NUP160	NUP188	NUP205	NUP214	NUP2L1	NUP358	NUP37	NUP43	NUP50	NUP53	NUP54	NUP58	NUP60	NUP62	NUP75	NUP88	NUP93	NUP96	POM121	POM152	RAE1	SEC13	SEH1
**At**	1			1	1		1		1	1	1		1	1	1	1	1	15			1	1	1	1	1		1	1	1	1	1			1	1	1
**Ce**			1		1	1	3		1	1	1		1	1		1	1		1		1	1	1	1			1	1	2	1	1			1	1	1
**Cr**	20			1			1		1	1	1		1	1		1					6		1	1	1		1	1	1	1	1			1	1	1
**Ct**			1	1		1	1	1	1	1			1	1	1	1	1	1						1	1		1	1	1	1	1		1	1	1	2
**Dm**	1		1	1	1	1	1		1	1	1	1	1	1	1	1	1		1	1	1	1	1	1	1		1	1	1	1	1			1	1	1
**Dr**	1	1	1	1	1	1	1		1	1	1	1	1	1	1	1	1	1	1	1	1	1	1	1	1		1	1	1	1	1	1		1	1	1
**Hs**	1	1	1	1	1	1	1		1	1	1	1	1	1	1	1	1	1	1	1	1	1	1	1	1		1	1	1	1	1	1		1	1	1
**Md**	1			1	2		1		2	1	1		1	1	1	1	1				1	1	2	1	1		1	1	1	1	1			1	1	1
**Mm**	1	1	1	1	1	1	1		1	1	1	1	1	1	1	1	1	1	1	1	1	1	1	1	1		1	1	1	1	1	1		1	1	3
**Mu**	1	1	1	1	1	1	1		1	1	1	1	1	1	1	1	1	1	1	1	1	1	1	1	1		1	1	1	1	1	1		1	1	1
**Sc**		1		1		1	1	1	1	1	1		2	1	1	1	1					1	1	1		1	1	1	1	1	1		1	1	1	1
**Sp**		2		1		1		1		1	1		1	1	1	1	1	1		24				1		1	1	1	1	1	1		1	1	1	1
**Tb**		1								1			1		1	1								1			1			1	1				1	1
**Tt**					1					2			1	17																1	1				3	1
**Xl**	1	1	1			1	1		1	1	1	1	1	1	1	1	1		1	1	1	1	1	1	1		1	1	1	1	1			1	1	1

Legend: At – Arabidopsis thaliana, Ce – Caenorhabditis elegans, Cr - Chlamydomonas reinhardtii, Ct - Chaetomium thermophilum, Dm – Drosophila melanogaster, Dr – Danio rerio, Hs – Homo sapiens, Md - Malus domestica, Mm – Macaca mulatta, Mu – Mus musculus, Sc – Saccharomyces cerevisiae, Sp - Schizosaccharomyces pombe, Tb – Trypanosoma brucei, Tt - Tetrahymena thermophila , Xl – Xenopus laevis

Supplementary Table 9. Position of the correct hit in the HMMSEARCH output with heuristic filters and masked sequences, using custom HMM profiles (NUP-HMM) based on collected sequences. Red-colored boxes refer to proteins that are not considered detected.

	AAAS	CENTRIN2	ELYS	GLE1	GP210	KRH2	MTOR	MUG31	NDC1	NUP107	NUP133	NUP153	NUP154	NUP160	NUP188	NUP205	NUP214	NUP2L1	NUP358	NUP37	NUP43	NUP50	NUP53	NUP54	NUP58	NUP60	NUP62	NUP75	NUP88	NUP93	NUP96	POM121	POM152	RAE1	SEC13	SEH1
**At**	1			1	1		1		1	1	1		1	1		1	1	15			1	1	1	1	1		1	1	2	1	1			1	1	1
**Ce**			1		1	1			1	1	1		1	1		1	1		1		1	1	1	1			1	1	1	1	1			1	1	1
**Cr**	20			1					1	1	1		1	1		1					6		1	1	1		1	1	1	1	1			1	1	1
**Ct**			1	1		1	1	1	1	1			1	1	1	1	1	1						1	1		1	1	1	1	1			1	1	1
**Dm**	1		1	1	1	1	1		1	1	1	1	1	1	1	1	1		1	1	1	1	1	1	1		1	1	1	1	1			1	1	1
**Dr**	1	2	1	1	1	1	1		1	1	1	1	1	1	1	1	1	1	1	1	1	1	1	1	1		1	1	1	1	1			1	1	1
**Hs**	1	1	1	1	1	1	1		1	1	1	1	1	1	1	1	1	1	1	1	1	1	1	1	1		1	1	1	1	1	1		1	1	1
**Md**	1			1	2		1		1		1		1	1		1	1				1	1	2	2	1		1	1	1	1	1			1	1	1
**Mm**	1	1	1	1	1	1	1		1	1	1	1	1	1	1	1	1	1	1	1	1	1	1	1	1		1	1	1	1	1	1		1	1	2
**Mu**	1	1	1	1	1	1	1		1	1	1	1	1	1	1	1	1	1	1	1	1	1	1	1	1		1	1	1	1	1	1		1	1	1
**Sc**		1		1		1	1	1	1	1	1		2	1		1						1	1	1		1	1	1	1	1	1		1	1	1	1
**Sp**		2		1		1		1		1	1		1	1	1	1	1	1		24			1			1	1	1	1	1	1		1	1	1	1
**Tb**		1								1			1		1	1								1			1			1	1				1	1
**Tt**					1								1	11																	1				3	1
**Xl**	1	1	1			1	1		1	1	1	1	1	1	1	1	1		1	1	1	1	1	1	1		1	1	1	1	1			1	1	1

Legend: At – Arabidopsis thaliana, Ce – Caenorhabditis elegans, Cr - Chlamydomonas reinhardtii, Ct - Chaetomium thermophilum, Dm – Drosophila melanogaster, Dr – Danio rerio, Hs – Homo sapiens, Md - Malus domestica, Mm – Macaca mulatta, Mu – Mus musculus, Sc – Saccharomyces cerevisiae, Sp - Schizosaccharomyces pombe, Tb – Trypanosoma brucei, Tt - Tetrahymena thermophila , Xl – Xenopus laevis

Supplementary Table 10. Detection of fungal species in an unknown dataset of MAGs. Detection of the fungal-specific nucleoporins was performed using PSSM profiles for Nup60, Mug31, POM152 and PFAM-HMM for Nup60. Y denotes at least one hit of the queried profiles against that bin, while N denotes not hits. Column "Fungal" denotes whether said bin refers to a fungal species; Y-Yes, N-No.

		PSSM		PFAM-HMM Heuristics off		PFAM-HMM Heuristics on	
MAG	Fungal	Masked	Unmasked	Masked	Unmasked	Masked	Unmasked
SRR13685148_bin.1	N	N	Y	N	Y	N	N
ERR2206775_bin.11	N	N	N	N	Y	N	N
SRR3997477_bin.32	N	N	Y	Y	Y	N	N
SRR1955887_bin.63	N	N	N	N	Y	N	N
SRR5821798_bin.148	N	N	Y	Y	Y	N	N
SRR13685159_bin.84	N	N	Y	Y	Y	N	N
ERR2206769_bin.121	N	N	N	Y	Y	N	N
ERR2206769_bin.70	N	N	Y	Y	Y	N	N
SRR13685149_bin.1	N	N	N	Y	Y	N	N
ERR868413_bin.18	N	N	Y	N	Y	N	N
ERR599023_bin.127	N	N	Y	N	Y	N	N
ERR598991_bin.40	N	N	Y	Y	Y	N	N
ERR2206775_bin.1	N	N	Y	Y	Y	N	N
ERR599097_bin.1	N	N	Y	Y	Y	N	N
ERR868478_bin.16	N	N	N	N	Y	N	N
ERR1726919_bin.5	N	N	Y	N	Y	N	N
ERR599298_bin.2	N	N	Y	Y	Y	N	N
ERR1430553_bin.42	N	N	Y	Y	Y	N	N
ERR868413_bin.0	N	N	Y	Y	Y	N	N
ERR912198_bin.19	N	N	Y	Y	Y	N	Y
ERR868508_bin.30	N	N	Y	Y	Y	N	N
ERR598983_bin.99	N	N	Y	N	Y	N	Y
ERR868366_bin.18	N	N	N	N	Y	N	N
ERR1305896_bin.110	N	N	Y	Y	Y	N	N
ERR868511_bin.18	N	N	Y	Y	Y	N	N
ERR1995252_bin.1	N	Y	Y	Y	Y	N	Y
ERR1094789_bin.1	Y	Y	Y	Y	Y	Y	Y
ERR1094797_bin.38	Y	Y	Y	N	Y	N	N
ERR1332600_bin.12	Y	Y	Y	Y	Y	N	N
ERR1332601_bin.1	Y	Y	Y	Y	Y	Y	Y
ERR4757804_bin.1	Y	Y	Y	Y	Y	Y	Y
ERR4757809_bin.16	Y	Y	Y	Y	Y	N	N
ERR4757809_bin.21	Y	Y	Y	Y	Y	Y	Y
ERR4757816_bin.32	Y	Y	Y	Y	Y	Y	Y
ERR4757829_bin.1	Y	Y	Y	Y	Y	Y	Y
ERR4757847_bin.1	Y	Y	Y	Y	Y	N	N
ERR4757856_bin.1	Y	Y	Y	Y	Y	Y	Y

ERR4757857_bin.32	Y	Y	Y	Y	Y	N	N
ERR599223_bin.4	Y	Y	Y	Y	Y	Y	Y
ERR868349_bin.39	Y	Y	Y	Y	Y	Y	Y
ERR868449_bin.23	Y	Y	Y	Y	Y	Y	Y
ERR868474_bin.13	Y	Y	Y	N	Y	N	N
SRR12240178_bin.23	Y	Y	Y	Y	Y	Y	Y
SRR12240181_bin.9	Y	Y	Y	Y	Y	Y	Y
SRR12240182_bin.18	Y	Y	Y	Y	Y	Y	Y
SRR12240183_bin.19	Y	Y	Y	Y	Y	Y	Y
SRR12240184_bin.7	Y	Y	Y	Y	Y	Y	Y
SRR12240185_bin.4	Y	Y	Y	Y	Y	Y	Y
SRR12395656_bin.4	Y	Y	Y	Y	Y	Y	Y
SRR13685122_bin.10	Y	Y	Y	Y	Y	Y	Y
SRR13685123_bin.11	Y	Y	Y	Y	Y	Y	Y
SRR13685124_bin.18	Y	Y	Y	Y	Y	Y	Y
SRR13685126_bin.1	Y	Y	Y	Y	Y	N	N
SRR13685127_bin.16	Y	Y	Y	Y	Y	Y	Y
SRR13685128_bin.34	Y	Y	Y	Y	Y	N	N
SRR13685129_bin.54	Y	Y	Y	Y	Y	Y	Y
SRR13685130_bin.1	Y	Y	Y	Y	Y	Y	Y
SRR13685131_bin.26	Y	Y	Y	Y	Y	Y	Y
SRR13685132_bin.23	Y	Y	Y	Y	Y	Y	Y
SRR13685134_bin.1	Y	Y	Y	Y	Y	Y	Y
SRR13685136_bin.1	Y	Y	Y	Y	Y	N	Y
SRR13685137_bin.4	Y	Y	Y	Y	Y	Y	Y
SRR13685138_bin.1	Y	Y	Y	Y	Y	Y	Y
SRR13685139_bin.2	Y	Y	Y	Y	Y	Y	Y
SRR13685140_bin.9	Y	Y	Y	Y	Y	Y	Y
SRR13685141_bin.8	Y	Y	Y	Y	Y	Y	Y
SRR13685143_bin.4	Y	Y	Y	Y	Y	Y	Y
SRR13685145_bin.0	Y	Y	Y	Y	Y	Y	Y
SRR13685147_bin.13	Y	Y	Y	Y	Y	Y	Y
SRR13685148_bin.33	Y	Y	Y	Y	Y	Y	Y
SRR13685149_bin.3	Y	Y	Y	Y	Y	Y	Y
SRR13685150_bin.2	Y	Y	Y	Y	Y	Y	Y
SRR13685152_bin.9	Y	Y	Y	Y	Y	Y	Y
SRR13685154_bin.12	Y	Y	Y	Y	Y	Y	Y
SRR13685155_bin.27	Y	Y	Y	Y	Y	Y	Y
SRR13685156_bin.6	Y	Y	Y	Y	Y	Y	Y
SRR13685157_bin.1	Y	Y	Y	Y	Y	Y	Y
SRR13685158_bin.1	Y	Y	Y	Y	Y	Y	Y
SRR13685159_bin.72	Y	Y	Y	Y	Y	N	N
SRR13685160_bin.30	Y	Y	Y	Y	Y	Y	Y
SRR13685162_bin.23	Y	Y	Y	Y	Y	N	N
SRR13685164_bin.3	Y	Y	Y	Y	Y	Y	Y
SRR13685165_bin.32	Y	Y	Y	N	Y	N	N

SRR13685166_bin.32	Y	Y	Y	Y	Y	Y	Y
SRR13685167_bin.0	Y	Y	Y	N	Y	N	N
SRR13685168_bin.22	Y	Y	Y	Y	Y	Y	Y
SRR13685169_bin.3	Y	Y	Y	Y	Y	Y	Y
SRR13685170_bin.17	Y	Y	Y	Y	Y	Y	Y
SRR13685171_bin.2	Y	Y	Y	Y	Y	Y	Y
SRR13685172_bin.10	Y	Y	Y	Y	Y	Y	Y
SRR13685173_bin.1	Y	Y	Y	Y	Y	Y	Y
SRR13685174_bin.6	Y	Y	Y	Y	Y	Y	Y
SRR13685175_bin.32	Y	Y	Y	Y	Y	Y	Y
SRR13685176_bin.20	Y	Y	Y	Y	Y	Y	Y
SRR13685177_bin.4	Y	Y	Y	Y	Y	Y	Y
SRR13685178_bin.5	Y	Y	Y	N	Y	N	N
SRR13685179_bin.16	Y	Y	Y	Y	Y	Y	Y
SRR13685180_bin.1	Y	Y	Y	Y	Y	Y	Y
SRR1562005_bin.67	Y	Y	Y	Y	Y	Y	Y
SRR1631526_bin.20	Y	Y	Y	Y	Y	N	N
SRR1632457_bin.10	Y	N	Y	N	Y	N	N
SRR1647000_bin.0	Y	Y	Y	Y	Y	N	N
SRR1647065_bin.29	Y	Y	Y	N	Y	N	N
SRR1779153_bin.70	Y	Y	Y	Y	Y	Y	Y
SRR3017086_bin.52	Y	Y	Y	Y	Y	Y	Y
SRR3017090_bin.1	Y	Y	Y	Y	Y	N	N
SRR3017090_bin.8	Y	Y	Y	Y	Y	N	Y
SRR3017092_bin.1	Y	Y	Y	Y	Y	N	Y
SRR3017095_bin.66	Y	Y	Y	Y	Y	N	N
SRR3017098_bin.23	Y	Y	Y	Y	Y	N	Y
SRR3017099_bin.44	Y	Y	Y	Y	Y	Y	Y
SRR3017103_bin.1	Y	Y	Y	Y	Y	Y	Y
SRR3017105_bin.41	Y	Y	Y	Y	Y	Y	Y
SRR3185024_bin.1	Y	Y	Y	Y	Y	N	N
SRR3546782_bin.2	Y	Y	Y	Y	Y	Y	Y
SRR3997477_bin.88	Y	Y	Y	Y	Y	Y	Y
SRR4028131_bin.1	Y	Y	Y	N	Y	N	Y
SRR4028141_bin.99	Y	Y	Y	Y	Y	Y	Y
SRR5438753_bin.5	Y	Y	Y	Y	Y	Y	Y
SRR6425773_bin.116	Y	Y	Y	Y	Y	Y	Y
SRR6853349_bin.25	Y	Y	Y	N	Y	N	N
SRR6853352_bin.14	Y	Y	Y	Y	Y	N	Y
SRR6853383_bin.36	Y	Y	Y	Y	Y	Y	Y
SRR7403881_bin.30	Y	Y	Y	Y	Y	Y	Y

Supplementary Table 11. Multiple sequence alignment "dimensions" for the nucleoporin families under the PSSM and profile-HMM models.

Nup	PSSM Alignment Length	NUP-HMM Alignment Length	NUP-PSSM & NUP-HMM Profiles - Number of sequences	Nf*	PSSM TP	PSSM FN	FNR-PSSM	NUP-HMM TP	NUP-HMM FN	FNR-NUP-HMM	Normalized Delta Length PSSM - NUP-HMM	Delta FNR PSSM - NUP-HMM
**aaas**	546	484	373	5.14	9	0	0.000	8	1	0.111	0.114	-0.111
**centrin2**	172	172	140	0.55	8	0	0.000	8	0	0.000	0.000	0.000
**elys**	2266	2235	372	2.82	7	1	0.125	8	0	0.000	0.014	0.125
**gle1**	677	549	516	9.79	11	0	0.000	11	0	0.000	0.189	0.000
**gp210**	1876	1831	631	4.12	8	2	0.200	8	2	0.200	0.024	0.000
**krh2**	276	247	547	12.59	10	0	0.000	10	0	0.000	0.105	0.000
**mtor**	2346	2255	462	3.40	12	1	0.077	12	1	0.077	0.039	0.000
**mug31**	229	199	207	7.74	3	0	0.000	3	0	0.000	0.131	0.000
**ndc1**	578	572	649	11.93	12	0	0.000	12	0	0.000	0.010	0.000
**nup107**	845	815	697	10.15	15	0	0.000	13	2	0.133	0.036	-0.133
**nup133**	1200	1154	611	8.10	12	2	0.143	13	1	0.071	0.038	0.071
**nup153**	1883	1495	398	2.80	7	3	0.300	6	4	0.400	0.206	-0.100
**nup154**	1365	1356	520	4.71	15	0	0.000	15	0	0.000	0.007	0.000
**nup160**	1411	1361	602	7.13	14	0	0.000	13	1	0.071	0.035	-0.071
**nup188**	1749	1699	290	1.49	12	1	0.077	13	0	0.000	0.029	0.077
**nup205**	2090	1993	545	4.41	14	0	0.000	14	0	0.000	0.046	0.000
**nup214**	1711	1694	656	7.32	12	2	0.143	12	2	0.143	0.010	0.000
**nup2l1**	423	421	288	6.38	7	3	0.300	6	4	0.400	0.005	-0.100
**nup358**	2695	2620	436	2.62	7	0	0.000	7	0	0.000	0.028	0.000
**nup37**	320	314	307	3.31	7	0	0.000	6	1	0.143	0.019	-0.143
**nup43**	358	354	346	6.31	10	0	0.000	9	1	0.100	0.011	-0.100

**nup50**	564	560	516	10.17	8	5	0.385	8	5	0.385	0.007	0.000
**nup53**	331	326	469	10.12	12	1	0.077	12	1	0.077	0.015	0.000
**nup54**	610	515	739	12.36	13	1	0.071	12	2	0.143	0.156	-0.071
**nup58**	546	473	536	5.58	9	4	0.308	10	3	0.231	0.134	0.077
**nup60**	736	554	63	1.03	2	0	0.000	2	0	0.000	0.247	0.000
**nup62**	394	382	609	18.72	14	1	0.067	14	1	0.067	0.030	0.000
**nup75**	668	635	658	10.56	14	0	0.000	14	0	0.000	0.049	0.000
**nup88**	702	693	591	9.65	13	1	0.071	13	1	0.071	0.013	0.000
**nup93**	796	794	525	4.92	15	0	0.000	15	0	0.000	0.003	0.000
**nup96**	1960	1938	898	7.16	15	0	0.000	15	0	0.000	0.011	0.000
**pom121**	1249	1087	383	3.66	4	1	0.200	4	1	0.200	0.130	0.000
**pom152**	1250	1227	331	4.48	3	0	0.000	3	0	0.000	0.018	0.000
**rae1**	346	341	577	5.69	13	0	0.000	13	0	0.000	0.014	0.000
**sec13**	356	303	700	8.46	15	0	0.000	14	1	0.067	0.149	-0.067
**seh1**	354	319	566	7.08	14	1	0.067	12	3	0.200	0.099	-0.133

**Nf is the effective number of sequences in a multiple sequence alignment calculated as described in (1)*
TP: Number of true positives, FN: Number of false negatives, FNR: False negative rate (=FN/(TP+FN))
Normalized delta length is calculated as: (PSSM Length – NUP-HMM length)/PSSM Length
Delta FNR is calculated as: FNR PSSM – FNR NUP-HMM

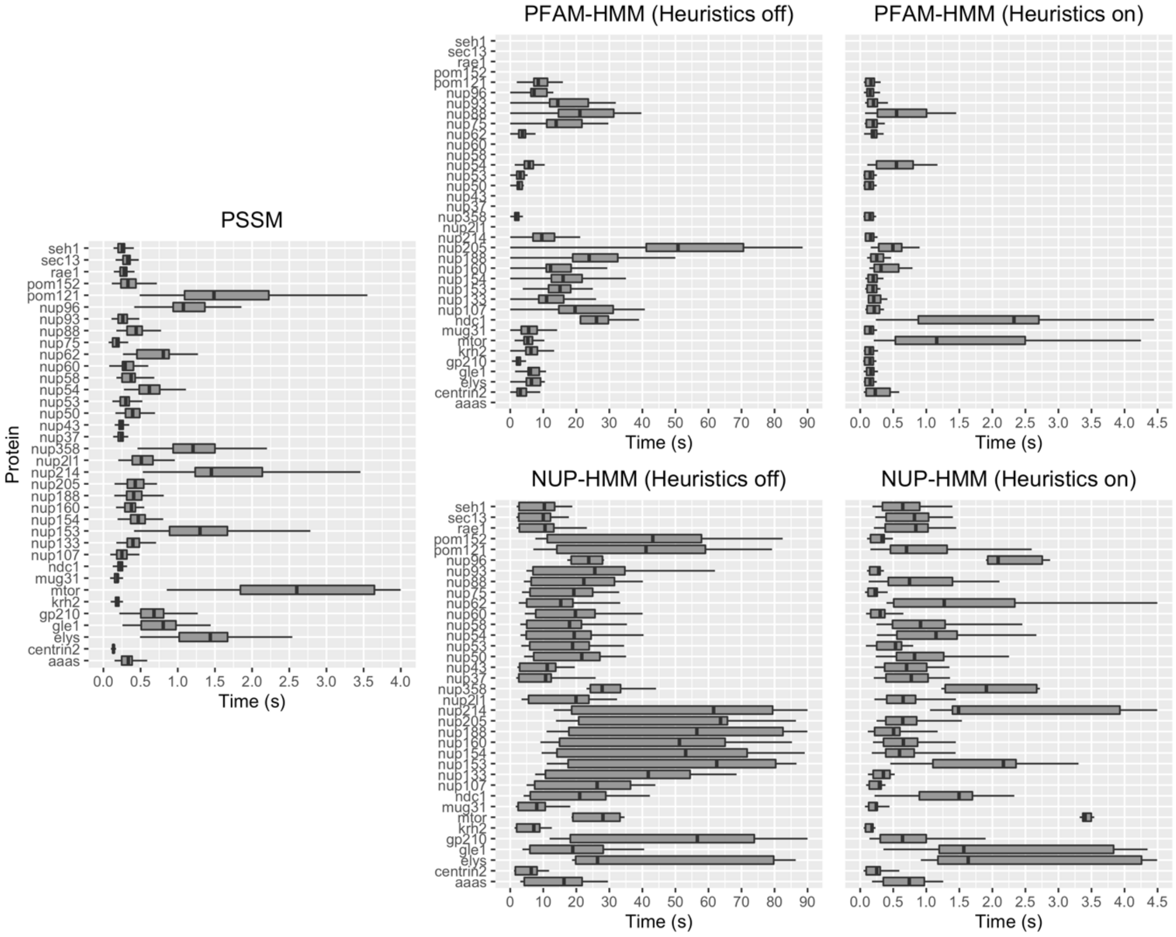

Supplementary Figure 1. Time required to query each profile against proteomes. The execution times were measured 10 times. PFAM-HMM profiles are shown only where applicable. PSSM querying is completed faster in comparison to PFAM-HMM with heuristic filters off. When heuristics filters are enabled, execution times for PFAM-HMM are similar to PSSM querying times, and in some cases faster. On the contrary, the NUP-HMM profiles present longer execution times.

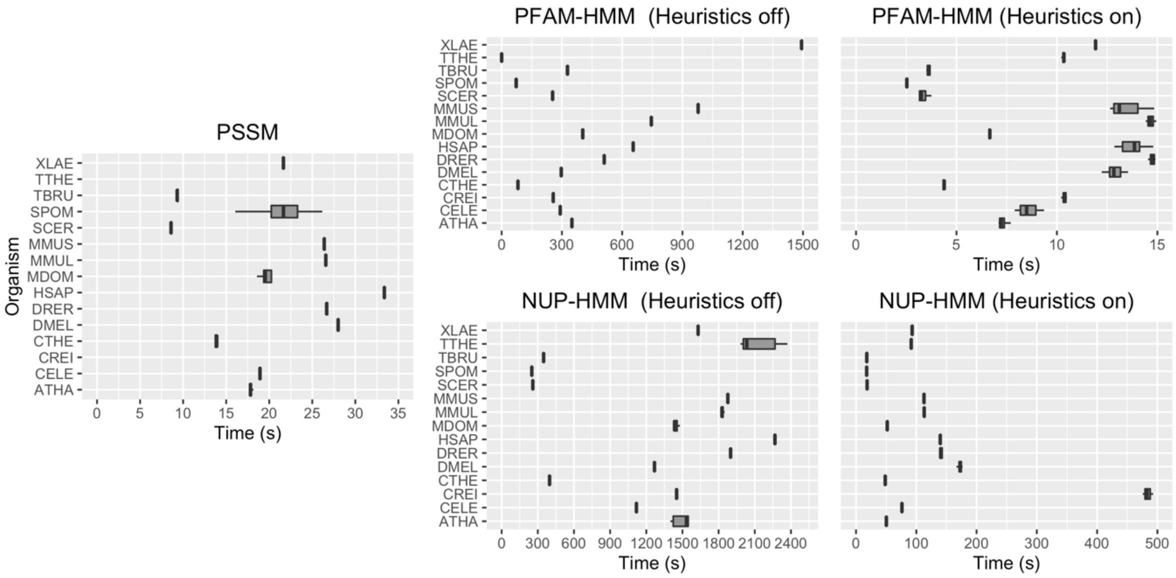

Supplementary Figure 2. Time required to query the whole set of profiles against proteomes. The execution times were measured 10 times. PSSM querying is completed faster in comparison to PFAM-HMM with heuristic filters off. When heuristics filters are enabled, execution times for PFAM-HMM is noticeably faster than PSSM. On the contrary, the NUP-HMM profiles present longer execution times.

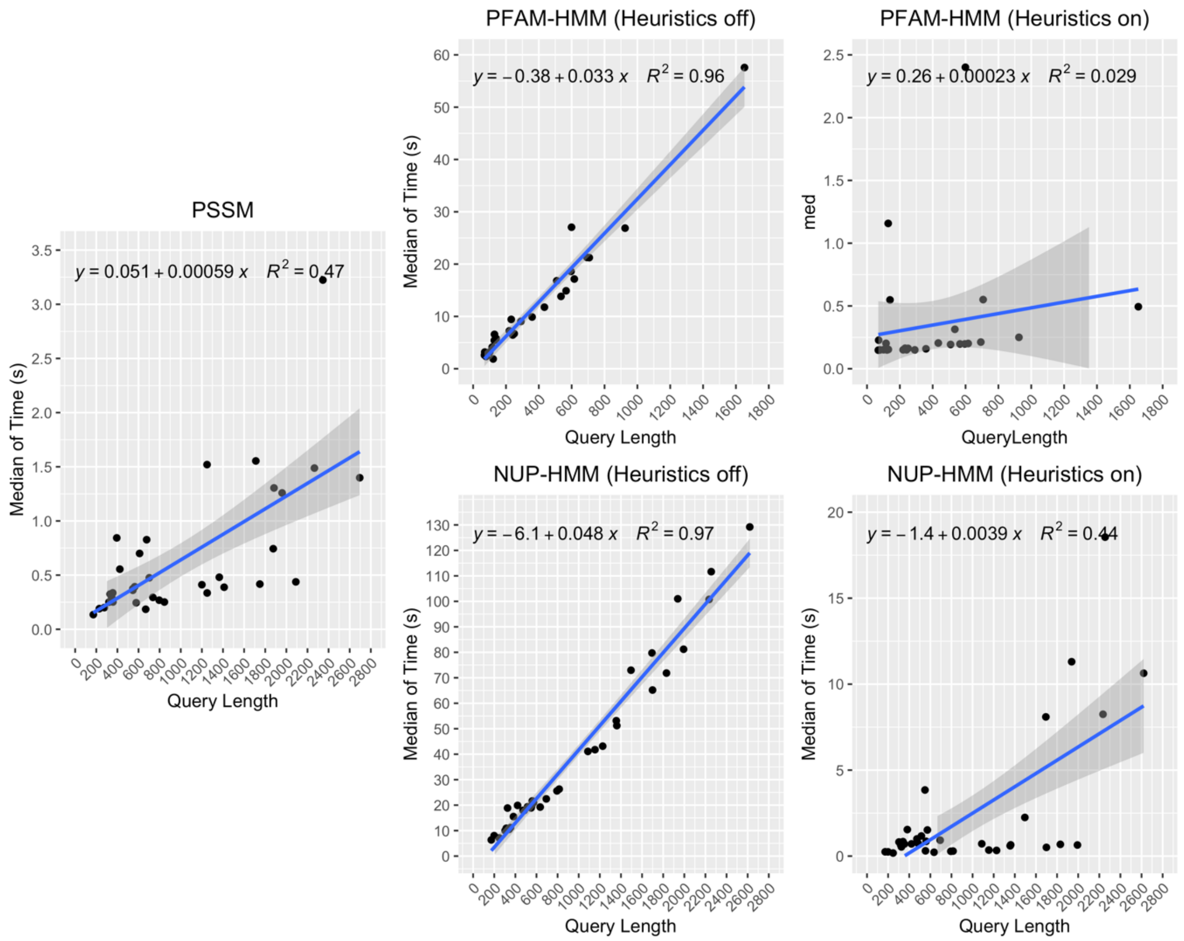

Supplementary Figure 3. Median time required to query the profiles, based on the query length (in aminoacids). Confidence interval is set to 0.95 (shown in grey shading). HMM profiles with heuristic filters turned off provide the least deviation from the trend line, something that's not observed when using heuristic filters. The latter, along with PSSM, present large deviations. Data available in GitHub repository.

References

1. Chengxin Zhang, Wei Zheng, S M Mortuza, Yang Li, Yang Zhang, DeepMSA: constructing deep multiple sequence alignment to improve contact prediction and fold-recognition for distant-homology proteins, Bioinformatics, Volume 36, Issue 7, 1 April 2020, Pages 2105–2112, https://doi.org/10.1093/bioinformatics/btz863.
